# Effects of bacterial fertilizer and green manure on soil enzyme activity and root characteristics in Korla fragrant pear orchard

**DOI:** 10.3389/fmicb.2025.1681490

**Published:** 2025-09-30

**Authors:** Zhanyi He, Jie Li, Lele Yang, Linsen Yan, Bolang Chen, Xing Shen, Zhongping Chai

**Affiliations:** ^1^College of Resources and Environment, Xinjiang Agricultural University, Urumqi, China; ^2^Xinjiang Key Laboratory of Soil and Plant Ecological Processes, Xinjiang Agricultural University, Urumqi, China

**Keywords:** Korla fragrant pear, green manure, soil nutrients, enzyme activity, root activity, yield

## Abstract

**Introduction:**

In arid regions, soil degradation and nutrient scarcity limit the productivity of Korla fragrant pear (*Pyrus bretschneideri Rehd.*). This study aimed to systematically evaluate how bacterial fertilizer and different green manures affect rhizosphere ecological functions and yield formation.

**Methods:**

A field experiment was conducted in a Korla pear orchard with six treatments: bacterial fertilizer (JF), two planting densities of sweet clover (CMX1, CMX2), two planting densities of oil sunflower (DK1, DK2), and a control (CK). Soil physicochemical properties, enzyme activities, root architecture, and yield were analyzed.

**Results:**

Both bacterial fertilizer and green manures significantly reduced soil pH and EC, improved nutrient content, and enhanced enzyme activity. Bacterial fertilizer was more effective in boosting enzyme activity, while sweet clover excelled in improving soil properties. Low-density green manures outperformed high-density ones. Root activity, vessel area, and yield were significantly increased by all amendments, with bacterial fertilizer showing the strongest effect. PLS-SEM analysis identified root activity as a key mediator linking soil improvements to yield gains.

**Discussion:**

These results highlight the critical role of root activity in translating soil amendments into yield benefits. Among all treatments, low-density sweet clover (CMX1) offers the most cost-effective and sustainable strategy for improving soil fertility and pear yield in arid orchards.

## 1 Introduction

**Figure 1:**
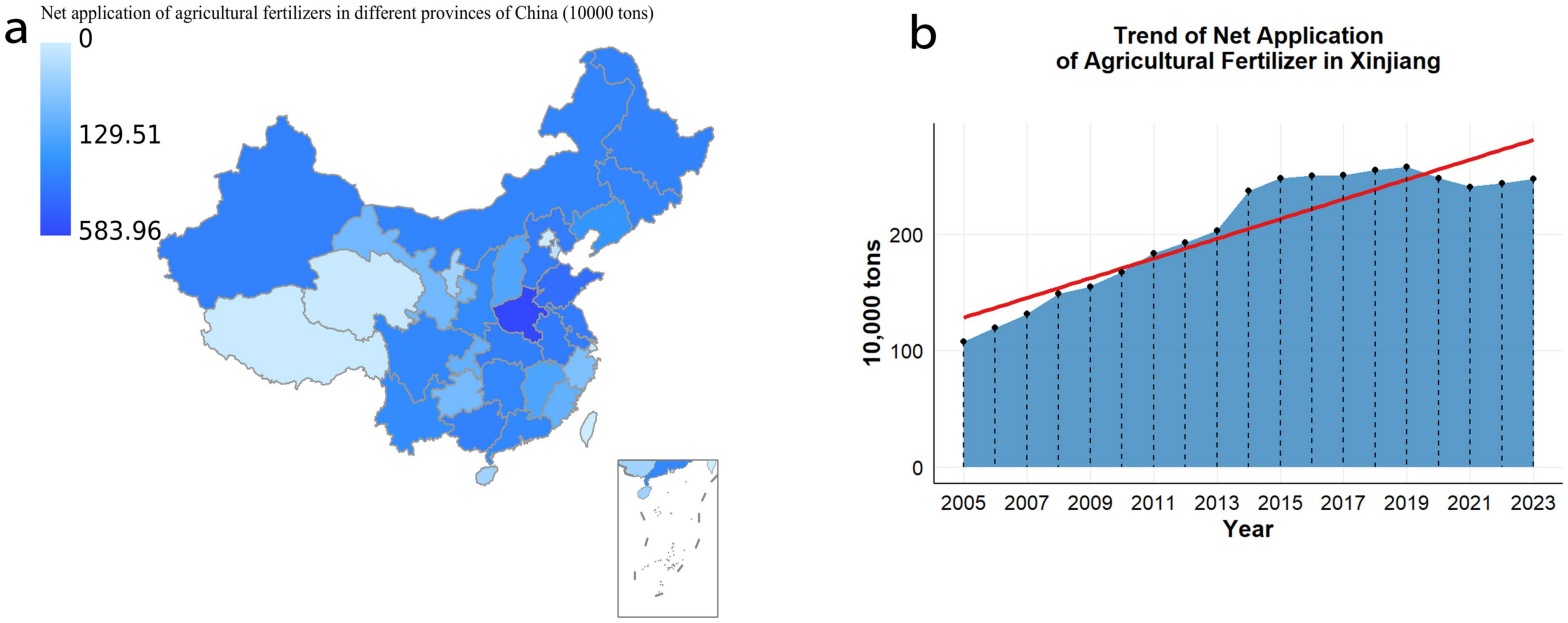
**(a)** In 2023, the National Bureau of Statistics applied the pure amount of agricultural chemical fertilizers in various regions of the country. **(b)** The amount of agricultural chemical fertilizer in Xinjiang from 2005 to 2023.

Fertilizers are recognized as essential inputs in modern agricultural production, providing crucial nutrients that promote healthy plant growth and improve soil fertility, ultimately leading to increased crop yields (Sorecha et al., 2025). As the global population rises and food demand continues to grow, the role of fertilizers in ensuring food security has become increasingly significant (Zhao et al., 2009). However, fertilizer application is a complex process, with the selection of appropriate types and methods exerting profound effects on agricultural outcomes. According to 2023 data from the National Bureau of Statistics, the standardized fertilizer application in Xinjiang reached 2.4766 million tons, ranking seventh nationwide (Figure 1a). From 2005 to 2023, fertilizer use in Xinjiang (Figure 1b) has increased substantially, intensifying concerns regarding soil acidification, compaction, and environmental pollution. These challenges threaten the stability of soil ecosystems and pose serious obstacles to sustainable agriculture (Mustafa et al., 2023). Improper fertilizer application may disrupt soil ecological balance, negatively affecting plant growth and soil health (Liu et al., 2021). Excessive use of chemical fertilizers suppresses soil microbial activity, destroys soil aggregate structure, and diminishes water and nutrient retention capacity (Kashyap et al., 2025). Furthermore, nutrient leaching from fertilizers contributes to water eutrophication and irreversible ecosystem damage (Ebhin Masto et al., 2006). Therefore, the development of scientific, environmentally friendly, and efficient fertilization practices is imperative for sustainable agriculture. In this context, the importance of bacterial fertilizer and green manure has become increasingly apparent. Bacterial fertilizer, rich in organic matter and microorganisms, effectively improve soil structure, enhance aeration and water retention, and provide sustained nutrient supply to crops. Green manure crops, through biological nitrogen fixation, convert atmospheric nitrogen into plant-available forms, thereby increasing soil nitrogen content (Lyu et al., 2024). Their root systems also promote soil structure, improve aeration and infiltration, reduce erosion, and suppress weed growth, benefiting subsequent crop yields (Cao et al., 2024; Krishnan et al., 1998). Compared to conventional chemical fertilizers, bacterial fertilizer and green manure generate lower carbon emissions and cause less environmental pollution, aligning with the principles of sustainable development (Min et al., 2023; Xiaomin et al., 2023). The pursuit of scientifically rational fertilizer application has thus become a critical focus in contemporary agricultural research and practice (He et al., 2024).

Korla fragrant pear, a significant economic crop in Xinjiang, has gained a strong reputation in the market due to its distinctive flavor and superior quality. Its cultivation not only provides substantial economic benefits for local farmers but also drives regional agricultural development (Wang et al., 2025). With a long history of cultivation, Korla pears are rich in vitamin C, dietary fiber, and various minerals, offering high nutritional value and health benefits, which are highly favored by consumers (Ouyang et al., 2018). However, the extended duration of cultivation and prolonged reliance on a single fertilization approach have gradually led to declining soil fertility in pear orchards, restricting root growth and ultimately affecting yields (Ding et al., 2019). Therefore, improving fertilization methods and optimizing fertilizer types, especially through the rational application of organic fertilizers, are considered crucial for enhancing soil quality in Korla pear orchards (Stumpf et al., 2025). This study focuses on the effects of different bacterial fertilizer and green manures on root–soil interactions in Korla pear orchards. By applying bacterial fertilizer, sweet clover, and oil sunflower green manure, the comprehensive impacts on soil fertility, physiological root indices, and pear yield are explored to provide scientific and practical guidance for the sustainable development of Korla pear orchards. It is anticipated that the findings will offer growers evidence based fertilization strategies, promote high-quality industry growth, and improve soil ecological conditions, serving as a reference for fertilization management in other economic fruit crops and supporting green, sustainable agricultural development.

## 2 Results

### 2.1 Effects of different bacterial fertilizer and green manures on soil physicochemical properties

**Figure 2:**
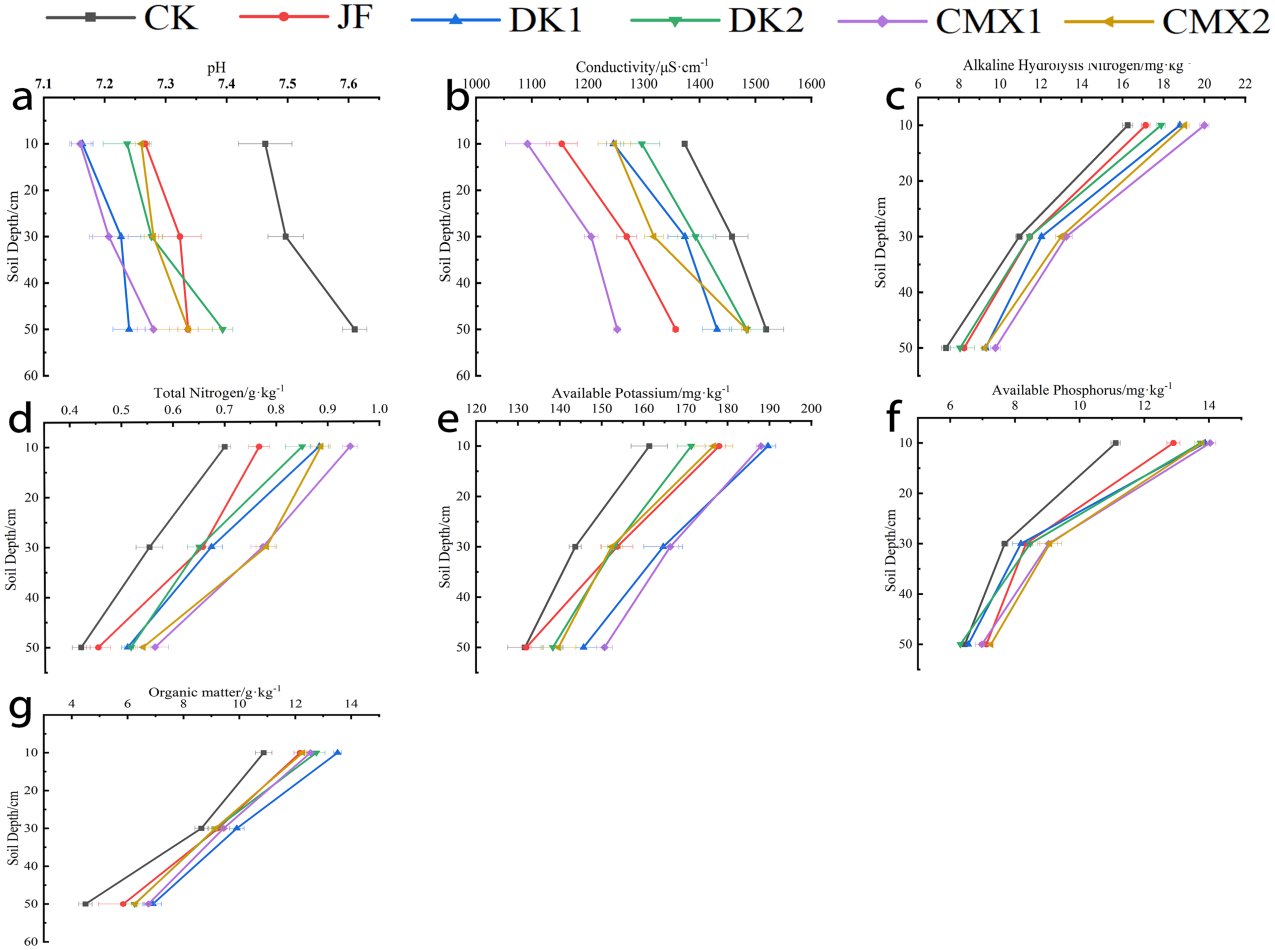
**(a–g)** The electric conductivity, pH, alkaline hydrolysis nitrogen, total nitrogen, available potassium, available phosphorus, and organic matter in the soil during the mature stage of Korla fragrant pear corresponded sequentially to the different bacterial fertilizer and green manure treatments.

As shown in Figure 2, significant differences (*p* < 0.05) were observed between all treatments and the CK control in the 0–20 cm soil layer for soil organic matter, total nitrogen, available nitrogen, and available phosphorus. Compared with CK, the organic matter content increased by 7.80, 16.95, 9.88, 8.90, and 6.37% in the JF, DK1, DK2, CMX1, and CMX2 treatments, respectively. Total nitrogen increased by 8.46, 20.40, 16.42, 31.34, and 25.87%, while available nitrogen increased by 18.66, 24.30, 22.65, 28.99, and 25.70%, and available phosphorus by 9.88, 19.74, 19.43, 18.37, and 17.78%, respectively. Available potassium was significantly higher (*p* < 0.05) in the JF, DK1, and CMX1 treatments, with increases of 7.50, 13.33, and 10.42%. Soil pH was significantly reduced (*p* < 0.05) in all treatments, with reductions of 1.44, 2.88, 1.57, 3.24, and 2.65%, respectively. Electrical conductivity was significantly lower under JF and CMX1 (*p* < 0.05), by 8.45 and 10.28%, respectively.

In the 20–40 cm soil layer, organic matter content in DK1 and CMX1 treatments was significantly higher than CK by 14.66 and 9.22% (*p* < 0.05). All treatments showed significantly higher total nitrogen content (*p* < 0.05), with increases of 18.67, 21.69, 17.47, 39.76, and 40.96%. Available nitrogen was significantly higher in DK1, DK2, CMX1, and CMX2 (*p* < 0.05), by 9.89, 4.57, 21.00, and 18.72%. Available phosphorus was significantly higher in DK2, CMX1, and CMX2, by 10.25, 17.68, and 18.14%. Significant increases (*p* < 0.05) in available potassium were seen in JF, DK1, DK2, and CMX1, by 6.96, 14.62, 6.50, and 15.78%. Soil pH was significantly reduced in all treatments, with reductions of 2.31, 3.60, 2.93, 3.87, and 2.89%. Electrical conductivity was significantly reduced in JF, DK1, CMX1, and CMX2, by 12.92, 5.78, 17.28, and 9.58%, respectively.

In the 40–60 cm layer, significant increases in organic matter and total nitrogen were observed in DK1, DK2, CMX1, and CMX2, with organic matter increasing by 53.64, 39.09, 50.15, and 39.39%, and total nitrogen by 21.43, 23.02, 34.13, and 28.57%. Available nitrogen was significantly higher in DK1, CMX1, and CMX2 (by 26.24, 32.81, and 25.57%). Available phosphorus was significantly higher in JF, CMX1, and CMX2 (by 10.33, 8.17, and 12.51%), while available potassium was significantly higher in DK1 and CMX1 (by 10.63 and 14.43%). Soil pH was significantly reduced in all treatments, with reductions of 3.59, 4.86, 2.85, 4.34, and 3.59%. Electrical conductivity was significantly reduced in JF, DK1, and CMX1, by 10.64, 5.77, and 17.53%, respectively.

Overall, JF treatment significantly increased soil organic matter, total nitrogen, available nitrogen, available phosphorus, and available potassium in the 0–20 cm layer, and reduced pH and electrical conductivity across 0–60 cm. Green manure treatments significantly increased soil organic matter, total nitrogen, available nitrogen, available phosphorus, and available potassium across 0–60 cm, while reducing soil pH and electrical conductivity. Oil sunflower was more effective than sweet clover in increasing organic matter, while sweet clover outperformed oil sunflower in improving available nutrients and reducing soil pH and conductivity, with CMX1 superior to CMX2.

### 2.2 Effects of different bacterial fertilizer and green manures on soil enzyme activities

**Figure 3:**
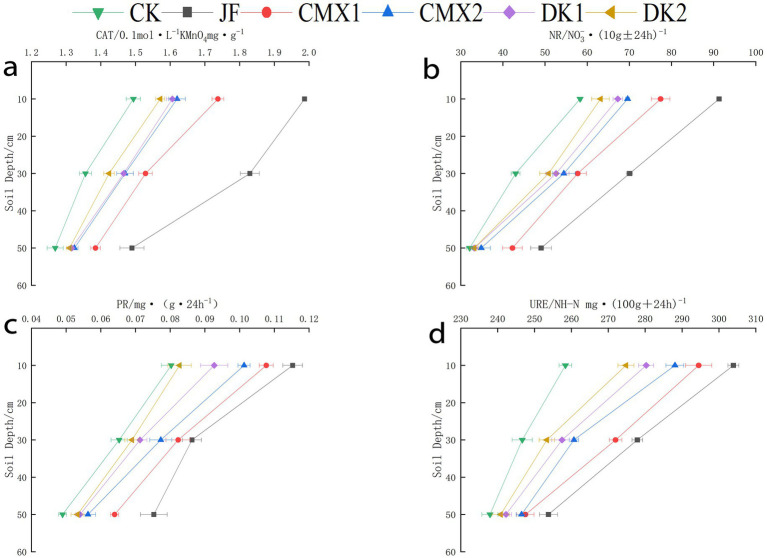
**(a–d)** Display the activities of soil protease, catalase, urease, and nitrate reductase during the mature period of Korla fragrant pear under different bacterial fertilizer and green manure treatments, respectively.

As shown in Figures 3a–d, the activities of urease, protease, catalase, and nitrate reductase decreased with increasing soil depth (0–60 cm). In the 0–20 cm layer, significant differences (*p* < 0.05) in soil nitrate reductase activity were observed for JF, DK1, CMX1, and CMX2 treatments compared to CK, with increases of 67.93, 15.23, 40.22, and 26.06%, respectively. Catalase activity was significantly higher in JF, CMX1, and CMX2 by 31.61, 9.54, and 5.20%, while protease activity increased by 41.35, 8.65, 34.62, and 22.12% in JF, DK1, CMX1, and CMX2, respectively. Urease activity was significantly enhanced by 12.03, 7.66, and 4.65% in JF, CMX1, and CMX2 treatments, respectively.

In the 20–40 cm layer, JF, DK1, DK2, CMX1, and CMX2 treatments significantly increased nitrate reductase activity by 67.68, 20.97, 18.72, 36.42, and 21.73%. Catalase activity was significantly improved in JF and CMX1 by 30.97 and 5.99%, while protease activity was higher in JF, CMX1, and CMX2 by 33.55, 27.74, and 16.77%. Urease activity increased significantly by 9.20, 4.35, 3.36, 8.75, and 4.06% under JF, DK1, DK2, CMX1, and CMX2, respectively.

In the 40–60 cm layer, JF and CMX1 treatments significantly enhanced nitrate reductase activity by 34.51 and 30.26%. Catalase activity was significantly higher only in JF (6.85%). Protease activity was significantly increased by 60.36, 27.03, 21.26, 45.95, and 33.33% in JF, DK1, DK2, CMX1, and CMX2, respectively. Urease activity was significantly improved by 6.39 and 2.39% in JF and CMX1.

Overall, compared to CK, application of JF and planting sweet clover as green manure significantly enhanced urease, protease, catalase, and nitrate reductase activities throughout the 0–60 cm soil profile, with the effect ranked as JF > CMX1 > CMX2. Sunflower green manure (DK1) had a pronounced effect on increasing nitrate reductase activity in the 0–40 cm layer and protease activity in the 40–60 cm layer, with DK1 > DK2.

### 2.3 Effects of different bacterial fertilizer and green manures on root activity of Korla fragrant pear

**Figure 4:**
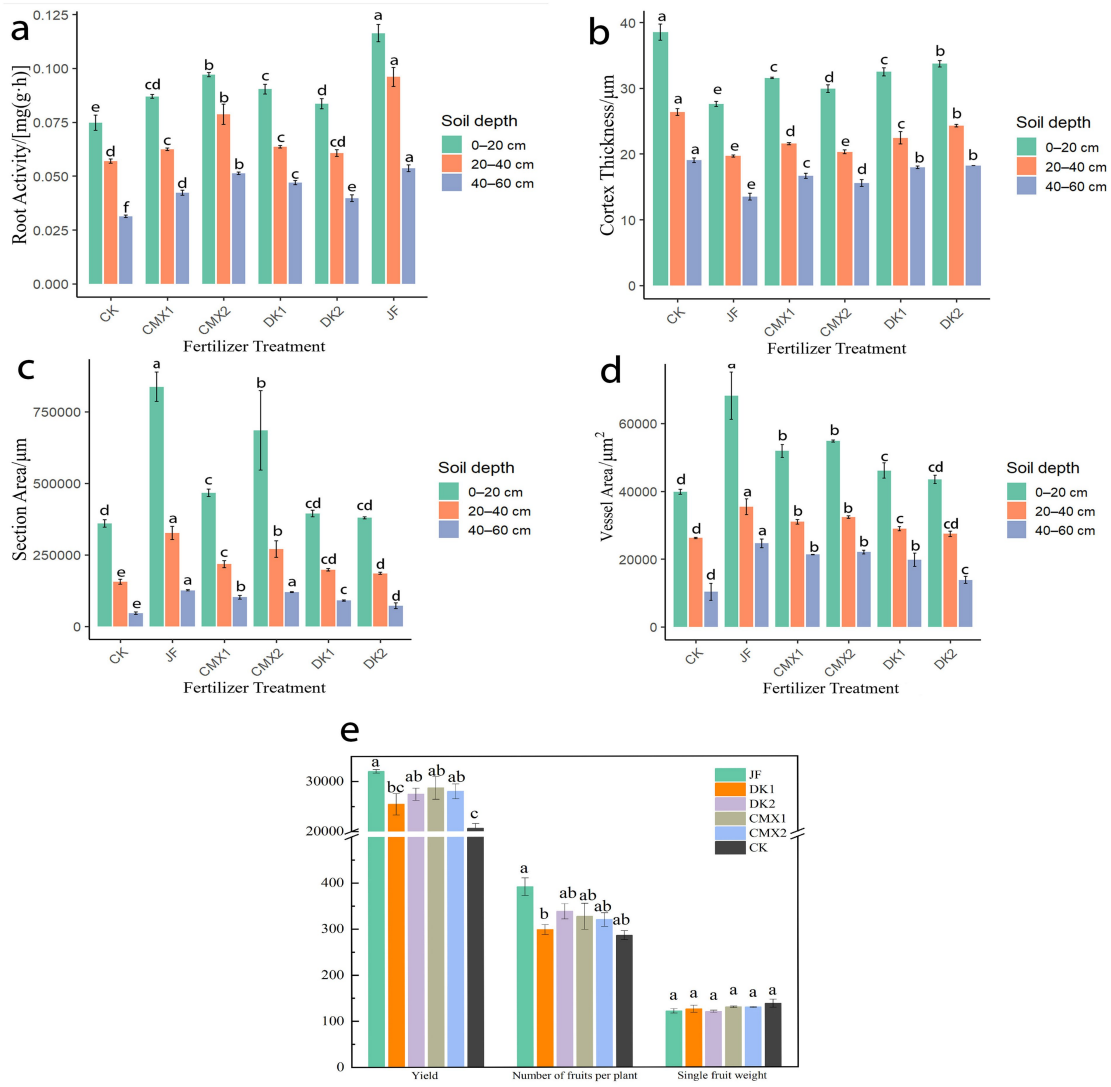
**(a)** Root activity of Korla fragrant pear at the mature stage under different bacterial fertilizer and green manure treatments. **(b–d)** Root cortex thickness, vessel area, and section area of the mature Korla fragrant pear under different bacterial fertilizer and green manure treatments, respectively. **(e)** Single fruit weight, yield per plant, and total yield of Korla fragrant pear under different bacterial fertilizer and green manure treatments.

As shown in Figure 4a, significant differences in root activity of Korla fragrant pear were observed among soil layers. Root activity declined with increasing soil depth (0–60 cm), reaching its maximum in the surface layer (0–20 cm). In the 0–20 cm layer, JF, CMX1, CMX2, DK1, and DK2 treatments resulted in significantly higher root activity than CK, with increases of 55.80, 16.43, 29.91, 20.98, and 11.88%, respectively. In the 20–40 cm layer, significant increases over CK were observed for JF (68.42%), CMX1 (9.36%), CMX2 (38.01%), and DK1 (11.70%). In the 40–60 cm layer, JF, CMX1, CMX2, DK1, and DK2 treatments significantly enhanced root activity by 71.28, 35.11, 63.83, 50.00, and 26.60%, respectively, compared with CK. Overall, both bacterial fertilizer and green manure applications significantly increased root activity in the soil profile, with the effect ranked as JF > CMX2 > DK1 > CMX1 > DK2.

### 2.4 Effects of different bacterial fertilizer and green manures on root activity of Korla fragrant pear

**Figure 5:**
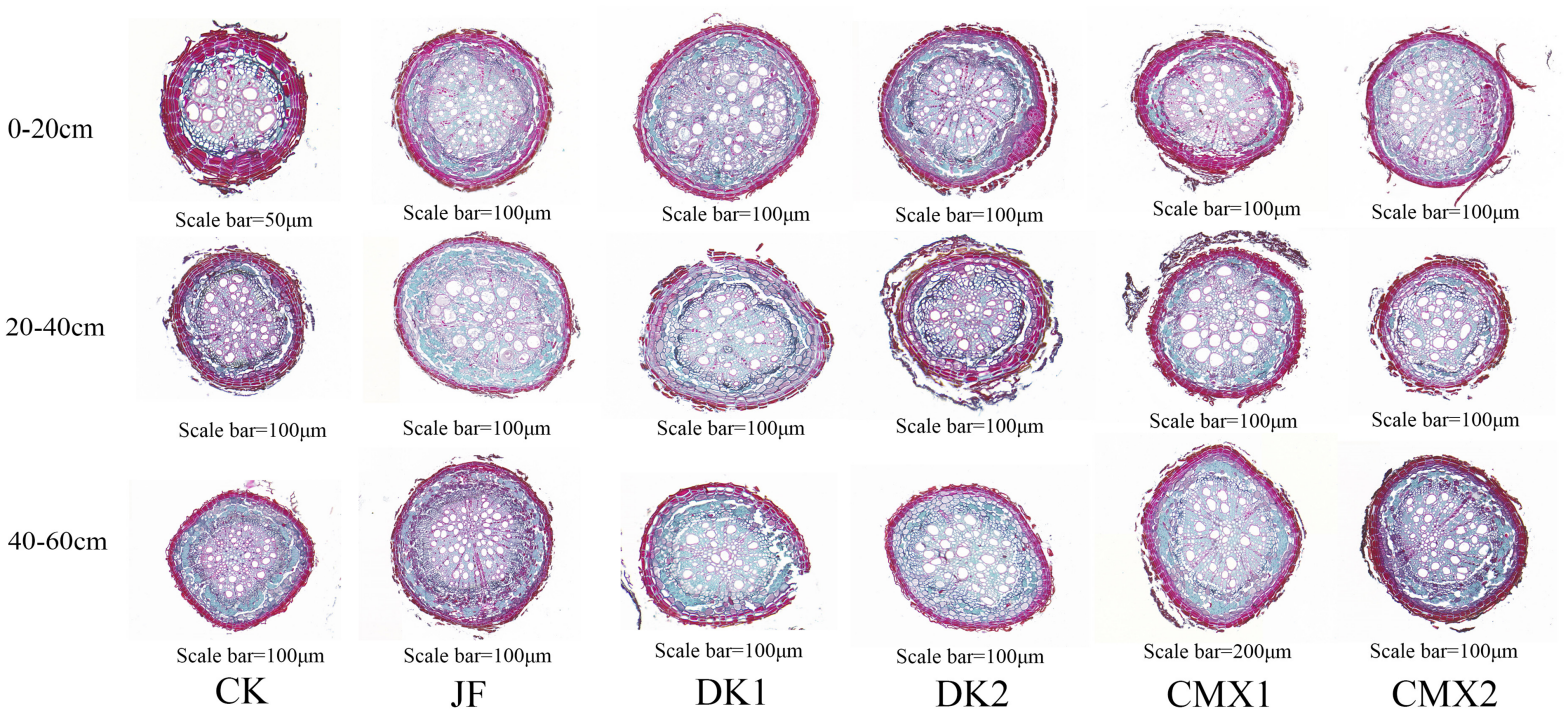
Paraffin sections of roots under different treatments.

As shown in Figure 5, the anatomical structure of the roots was primarily assessed by examining indicators related to cortex thickness, the proportions of various cell types within the xylem and phloem, vessel area, and section area.

#### 2.4.1 Effects of different bacterial fertilizer and green manures on root cortex thickness

As shown in Figure 4b, both bacterial fertilizer and green manure treatments reduced root cortex thickness compared with the CK control. In the 0–20 cm soil layer, JF, CMX1, CMX2, DK1, and DK2 treatments significantly decreased root cortex thickness by 28.4, 18.05, 22.37, 15.66, and 12.50%, respectively (*p* < 0.05). In the 20–40 cm layer, significant reductions of 25.28, 18.19, 22.93, 14.79, and 7.74% were observed for JF, CMX1, CMX2, DK1, and DK2 (*p* < 0.05). In the 40–60 cm layer, the corresponding reductions were 28.99, 12.45, 18.16, 5.54, and 4.20% (*p* < 0.05). Overall, both bacterial fertilizer application and green manure planting effectively reduced root cortex thickness compared to CK, with bacterial fertilizer showing greater effectiveness than green manure. Among green manure treatments, the decreasing effect followed the order: CMX2 > CMX1 > DK1 > DK2.

#### 2.4.2 Effects of different bacterial fertilizer and green manures on the proportion of cellulose cell wall cells in xylem

**Figure 6:**
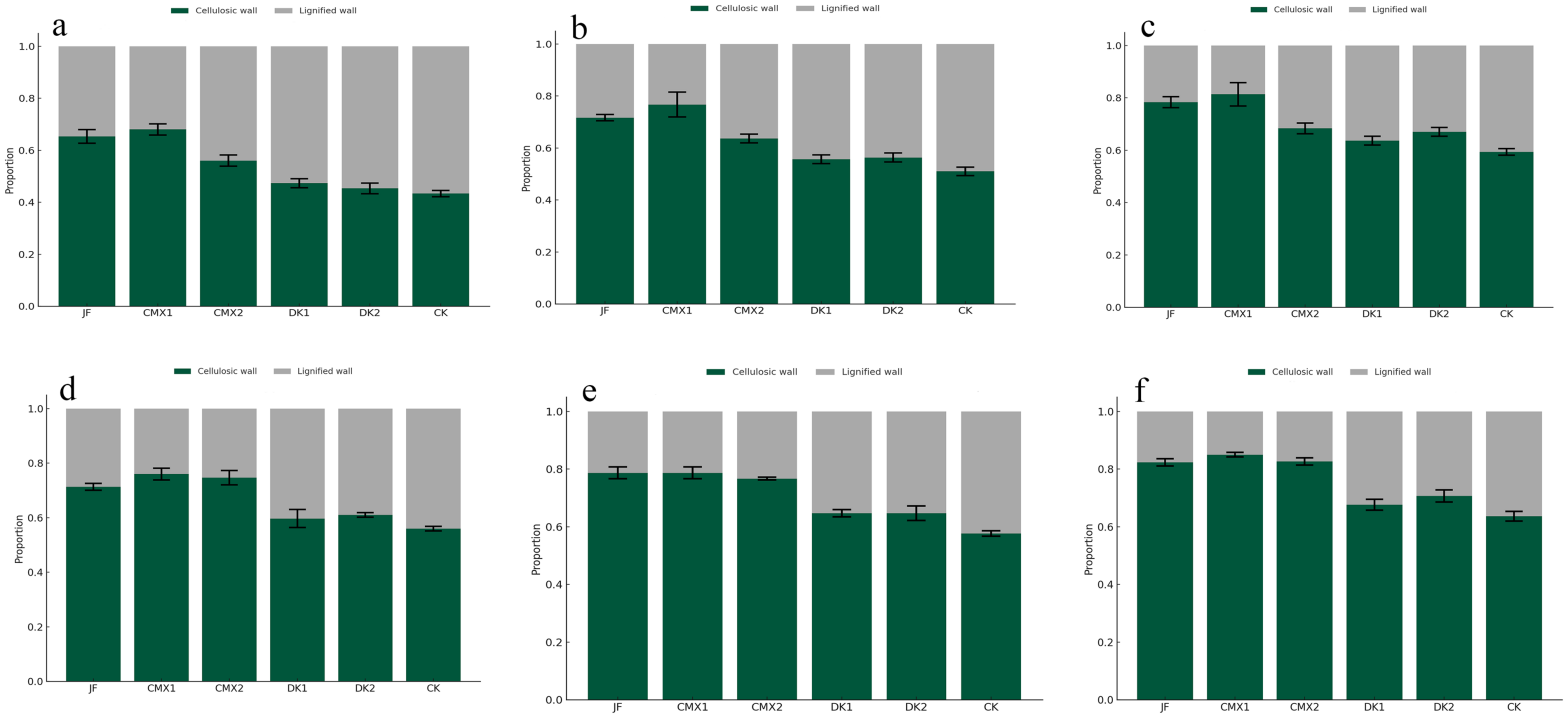
**(a–c)** Show the proportion of cellulose cell wall cells in the xylem at 0–20, 20–40, and 40–60 cm soil depths, respectively; **(d–f)** present the proportion of cellulose cell wall cells in the phloem at 0–20, 20–40, and 40–60 cm soil depths.

As shown in Figures 6a–c, both sweet clover green manure and bacterial fertilizer significantly increased the proportion of cellulose cell wall cells in xylem, and this proportion gradually increased with soil depth. In the 0–20 cm soil layer, the proportion of cellulose cell wall cells in xylem in the JF, CMX1, and CMX2 treatments was 65.67, 68.07, and 56.64%, respectively, which represented significant increases of 21.52, 23.92, and 12.49% compared to CK (*p* < 0.05). In the 20–40 cm layer, JF, CMX1, and CMX2 treatments showed proportions of 72.16, 77.85, and 64.09%, corresponding to increases of 20.69, 26.38, and 12.62% over CK (*p* < 0.05). In the 40–60 cm layer, the proportion reached 78.87% (JF), 81.71% (CMX1), and 68.36% (CMX2), significantly higher than CK by 19.16, 22.00, and 8.65%, respectively (*p* < 0.05). Overall, the proportion of cellulose cell wall cells in xylem increased with soil depth in all treatments, with CMX1 and JF showing the most pronounced effects, followed by CMX2. No significant difference was observed between DK treatments and CK.

#### 2.4.3 Effects of different bacterial fertilizer and green manures on the proportion of cellulose cell wall cells in phloem

As shown in Figures 6d–f, both sweet clover green manure and bacterial fertilizer treatments significantly increased the proportion of cellulose cell wall cells in phloem, with this proportion progressively increasing with soil depth. In the 0–20 cm soil layer, JF, CMX1, and CMX2 treatments resulted in phloem proportions of 71.85, 73.78, and 78.00%, representing significant increases of 15.76, 17.69, and 21.91% over CK (*p* < 0.05). In the 20–40 cm layer, JF, CMX1, CMX2, and DK2 treatments reached 79.76, 79.59, 77.47, and 69.80%, with significant increases of 21.03, 20.86, 18.74, and 11.07% compared to CK (*p* < 0.05). In the 40–60 cm layer, the corresponding values were 83.17% (JF), 83.49% (CMX1), 83.20% (CMX2), 72.13% (DK1), and 71.72% (DK2), which were significantly higher than CK by 19.20, 18.91, 18.88, 7.84, and 7.43% (*p* < 0.05), respectively. Overall, the proportion of cellulose cell wall cells in phloem increased with soil depth, and sweet clover and bacterial fertilizer treatments demonstrated the most significant promotive effects, especially for JF, CMX1, and CMX2, while DK treatments had weaker effects.

#### 2.4.4 Effects of different bacterial fertilizer and green manures on vessel area and section area

As shown in Figure 4c, both bacterial fertilizer and green manure treatments significantly increased root vessel area compared to CK. In the 0–20 cm soil layer, JF, CMX1, CMX2, and DK1 treatments resulted in vessel area increases of 71.31, 30.29, 37.63, and 15.77% over CK, respectively (*p* < 0.05). In the 20–40 cm layer, vessel area was significantly increased by 35.03% (JF), 18.17% (CMX1), 23.33% (CMX2), and 10.43% (DK1) compared to CK (*p* < 0.05). In the 40–60 cm layer, all treatments significantly enhanced vessel area, with increases of 138.32, 106.38, 113.33, 91.12, and 34.05% (JF, CMX1, CMX2, DK1, and DK2, respectively; *p* < 0.05).

As shown in Figure 4d, root section area was also significantly increased by bacterial fertilizer and green manure. In the 0–20 cm layer, JF, CMX1, and CMX2 increased section area by 132.98, 29.90, and 90.58%, respectively, compared to CK (*p* < 0.05). In the 20–40 cm layer, all treatments showed significant increases over CK by 108.90, 39.16, 72.63, 26.66, and 18.37% (JF, CMX1, CMX2, DK1, and DK2, respectively; *p* < 0.05). In the 40–60 cm layer, section area increased by 175.02, 122.23, 162.14, 97.01, and 58.56% under JF, CMX1, CMX2, DK1, and DK2, respectively, all significantly greater than CK (*p* < 0.05).

Overall, both bacterial fertilizer and green manure significantly enhanced the vessel area and section area of Korla fragrant pear roots, with bacterial fertilizer having the strongest effect, followed by green manures in the order CMX2 > CMX1 > DK1 > DK2.

### 2.5 Principal component analysis based on soil physicochemical properties, enzyme activities, and root indices

**Figure 7:**
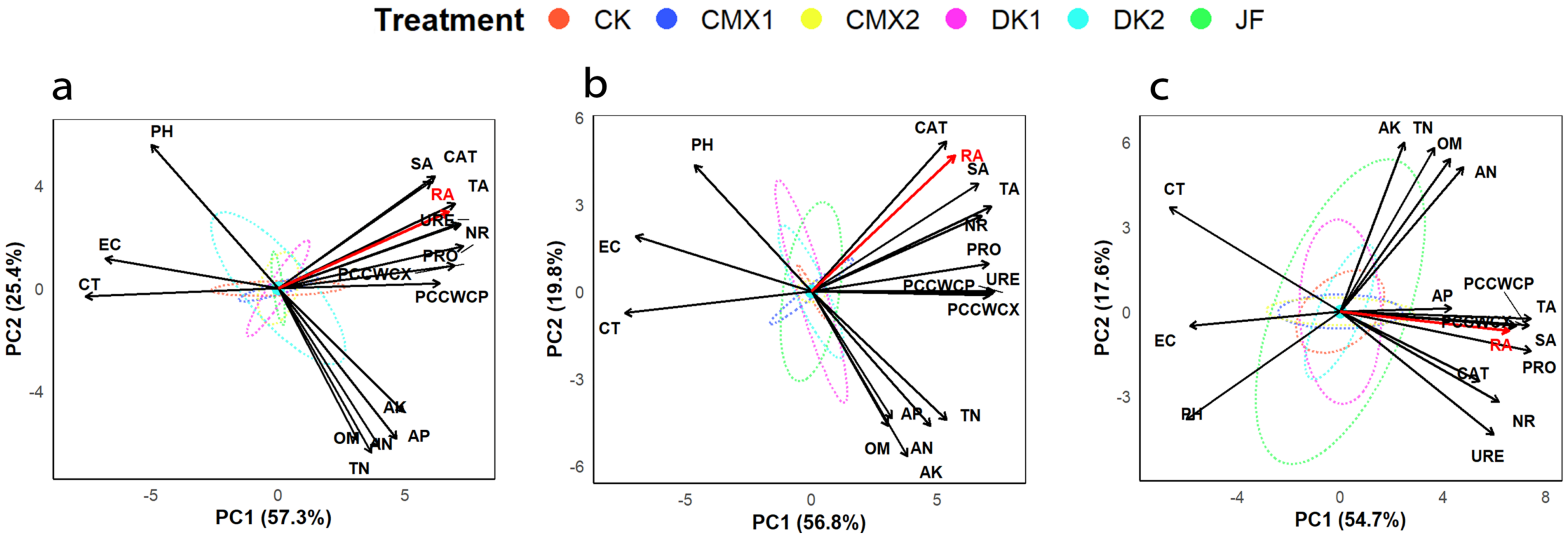
**(a–c)** From a to c are the principal component analyses based on soil physicochemical properties, enzyme activities, and root indicators for the 0–20 cm, 20–40 cm, and 40–60 cm soil layers, respectively.

According to the principal component analysis results (Figure 7), the first two principal components (PC1 and PC2) explained 82.7, 76.6, and 72.3% of the total variance in the 0–20 cm, 20–40 cm, and 40–60 cm soil layers, respectively, under different bacterial fertilizer and green manure treatments, effectively accounting for the overall variability in root activity related indices. The contribution rates of PC1 in the three soil layers were 57.3, 56.8, and 54.7%, respectively, mainly driven by root section indices (proportion of cellulose cell wall cells in xylem and phloem, cortex thickness, vessel area, section area) and soil enzyme activities (protease, urease, nitrate reductase, and catalase), representing root structural and enzymatic characteristics and serving as the core factors influencing root activity. PC2 contributed 25.4, 19.8, and 17.6% in the three layers, primarily influenced by soil physicochemical properties. Root activity was strongly positively correlated with PC1 across all soil layers, further confirming the key roles of root anatomical structure and enzyme activity in enhancing root activity. In contrast, root activity exhibited a weak positive correlation with PC2 in the surface and middle layers but shifted to a weak negative correlation in the deep layer, indicating that soil physicochemical properties have a certain regulatory effect on root activity.

Comprehensive analysis revealed that vessel area, section area, the proportion of cellulose cell wall cells in xylem and phloem, and soil enzyme activities were all significantly positively correlated with root activity, while cortex thickness, electrical conductivity, and pH showed significant negative correlations with root activity. Soil nutrient content was weakly positively correlated with root activity.

### 2.6 Effects of different bacterial fertilizer and green manures on the yield of Korla fragrant pear

As shown in Figure 4e, both bacterial fertilizer and green manure treatments had a significant impact on the yield of Korla fragrant pear. There were no significant differences among treatments in either fruit number per tree or single fruit weight. However, total yield was significantly higher for all bacterial fertilizer and green manure treatments compared to the control (CK). Specifically, JF, CMX1, CMX2, and DK2 treatments increased total yield by 55.3, 39.3, 35.9, and 33.2% relative to CK (*p <* 0.05). Although JF achieved the greatest yield increase, no significant differences in yield were observed among JF, CMX1, CMX2, and DK2.

### 2.7 Correlations among soil nutrients, enzyme activities, root activity, and yield under different bacterial fertilizer and green manure treatments

**Figure 8:**
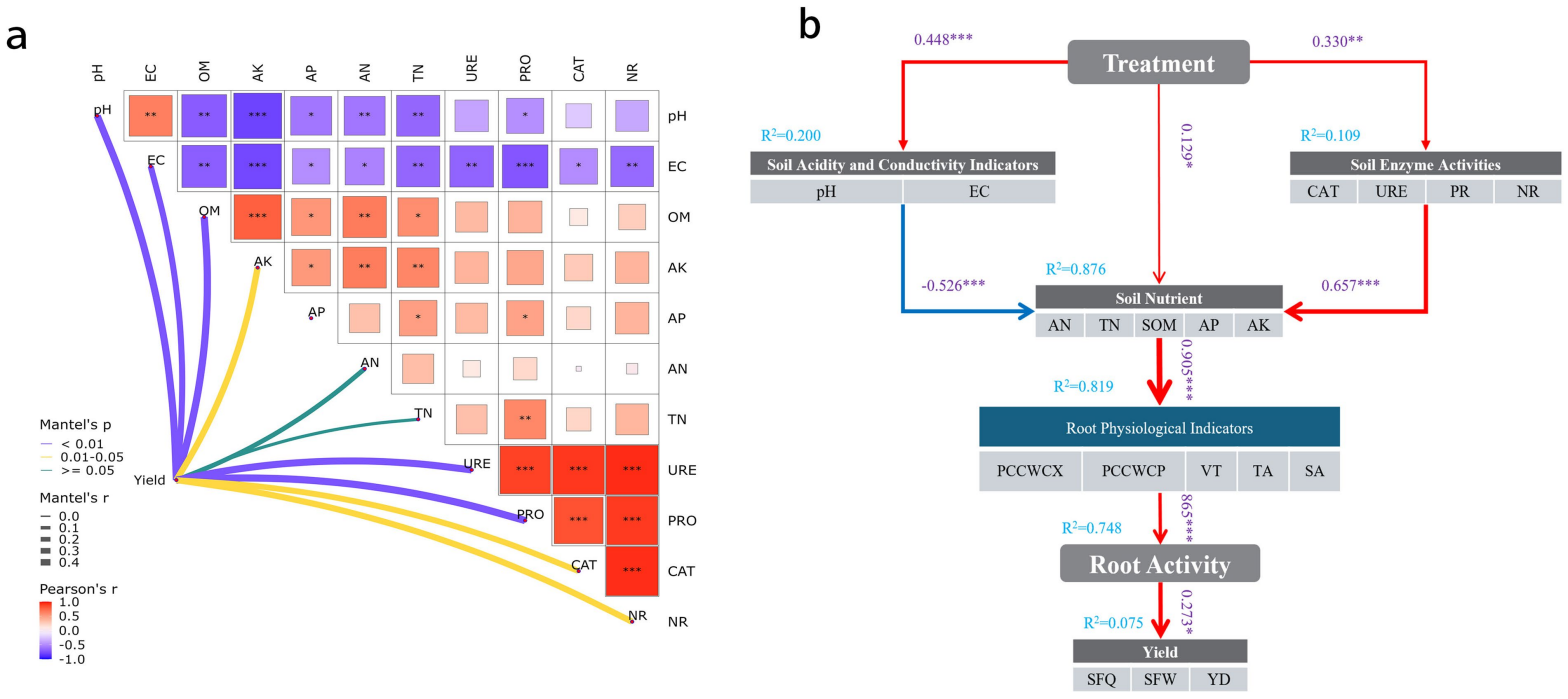
**(a)** Correlations among soil physicochemical properties, enzyme activities, root activity, and yield under different bacterial fertilizer and green manure treatments. **(b)** PLS-SEM path analysis diagram (*p < 0.05; **p < 0.01; ***p < 0.001).

To investigate the influence of soil physicochemical properties and enzyme activities on crop yield, Mantel tests combined with Pearson correlation analysis were conducted, as presented in Figure 8a. The results revealed significant correlations among soil properties such as pH, electrical conductivity (EC), and organic matter (OM), as well as strong associations between these physicochemical parameters and enzyme activities (URE, PRO, CAT, NR). This suggests that changes in the soil physicochemical environment may impact soil microbial activity and enzyme expression. Mantel’s test further indicated that soil pH, EC, OM, available potassium (AK), and various enzyme activities were all significantly correlated with crop yield. These findings suggest that managing soil physicochemical properties and improving soil enzyme activity can help enhance yield in practical agricultural production. Enzyme activities were also found to be highly positively intercorrelated, indicating a synergistic response within the soil enzyme system. Moreover, these enzyme activities were closely related to crop yield.

To further elucidate the potential pathways by which fertilization affects crop yield, a structural equation model based on partial least squares path modeling (PLS-SEM) was constructed (Figure 8b), systematically analyzing the indirect effects of fertilization on yield via soil physicochemical properties, enzyme activities, and root physiological characteristics. The modeling results demonstrated that fertilization treatments significantly improved soil chemical properties (path coefficient = 0.448, *p <* 0.001), soil nutrient levels (path coefficient = 0.129, *p <* 0.05), and soil enzyme activities (path coefficient = 0.330, *p <* 0.01), highlighting the positive role of fertilization in optimizing the soil environment. Soil pH and EC exhibited a negative correlation with soil nutrient levels (path coefficient = −0.526, *p <* 0.001). Soil enzyme activity significantly enhanced soil nutrient accumulation (path coefficient = 0.657, *p <* 0.001), reflecting the key role of enzymatic processes in nutrient transformation. Soil nutrients significantly promoted root physiological indices (path coefficient = 0.905, *p <* 0.001) and subsequently drove increases in root activity (path coefficient = 0.865, *p <* 0.001). Root activity, as a direct factor, contributed positively to crop yield (path coefficient = 0.273, *p <* 0.05).

## 3 Discussion

### 3.1 Effects of different bacterial fertilizer and green manures on soil physicochemical properties

The results of this study indicate that application of JF significantly increased soil organic matter, total nitrogen, alkaline hydrolysis nitrogen, available phosphorus, and available potassium in the 0–20 cm soil layer, while reducing soil pH and electrical conductivity (EC) throughout the 0–60 cm profile. Research by Wang et al. (2025) and Yodphet et al. (2025) has shown that bacterial fertilizer enhance soil organic matter content and structural stability through the synergistic action of diverse functional microorganisms, which decompose soil organic residues and convert them into stable organic matter. Nitrogen-fixing bacteria in bacterial fertilizer can fix atmospheric nitrogen into forms available to plants, while microbial metabolism produces organic acids that help neutralize soil alkalinity, decrease pH, and promote the decomposition and transformation of salts, thus reducing salt accumulation and lowering EC. Collectively, these effects improve soil fertility and the overall environment, creating more favorable conditions for plant growth.

Similarly, the use of green manure significantly increased soil organic matter, total nitrogen, alkaline hydrolysis nitrogen, available phosphorus, and available potassium across the 0–60 cm soil layer, while decreasing pH and EC. According to Lan et al. (2014), green manure crops fix large amounts of carbon through photosynthesis during growth, and after being incorporated into the soil at maturity, their decomposition by soil microbes releases abundant nutrients, thereby improving soil physicochemical properties. Studies by Al-Makhlof et al. (2022) and Huang et al. (2025) have further shown that oil sunflower green manure, due to its higher biomass and faster decomposition rate compared to sweet clover, results in greater improvements in soil organic matter. The two density levels in this study were selected according to local agronomic practice for green manure management in Korla fragrant pear orchards. Although the gradient was limited, these settings represent typical field configurations and therefore provide meaningful insights into their practical applicability. Future studies with finer density gradients and independent variation of row spacing will help clarify potential non-linear density–yield relationships.

In contrast, sweet clover outperformed oil sunflower in increasing rapidly available nutrients and reducing soil pH and EC. Dakora and Phillips (2002) and Drinkwater et al. (1998) reported that organic acids such as oxalic acid and citric acid secreted by sweet clover roots can chelate soil metal ions, release immobilized phosphorus, and neutralize some alkaline and saline components, thereby lowering soil pH. Legume-based cropping systems also help reduce nutrient loss and salt accumulation, enhancing overall soil health.

### 3.2 Effects of different bacterial fertilizer and green manures on soil enzyme activities

In this study, all bacterial fertilizer and green manure treatments significantly affected soil enzyme activities to varying degrees. Overall, the activities of urease, protease, catalase, and nitrate reductase were elevated under bacterial fertilizer and green manure treatments, with the most pronounced increases observed in the 0–20 cm surface soil. According to Shen et al. (2021), bacterial fertilizer are rich in diverse functional microorganisms such as denitrifying bacteria and proteolytic bacteria, which can significantly stimulate soil nitrogen transformation and organic matter decomposition, thereby enhancing various soil enzyme activities. In the present experiment, bacterial fertilizer (JF) increased the activity of all measured soil enzymes, particularly nitrate reductase and protease, which were elevated by 67.93 and 41.35% compared to the control, respectively.

Regarding green manure treatments, Rao et al. (2021) reported that plant residues and root activity can improve the soil microecological environment and thus promote various enzyme activities, a finding corroborated by the results for CMX1 and CMX2 in this study. As soil depth increased (20–40 cm), the promoting effects of all treatments on enzyme activities remained evident, though the overall magnitude declined compared to the surface. Xu et al. (2023) suggested that such effects extending into deeper soil layers may be attributed to the downward migration of watersoluble organic matter and root penetration.

In the 40–60 cm deep soil, the effects of bacterial fertilizer and green manure treatments diminished, with only JF and CMX1 maintaining significant enhancement of most enzyme activities. Amadou et al. (2020) indicated that the sustained release of bacterial fertilizer can activate the microecosystem in deeper soil layers. Both bacterial fertilizer and sweet clover green manure not only significantly improved surface soil enzyme activities, but also contributed to the functional enhancement of deep soil microbial communities. These results are consistent with findings by Carlson et al. (2015) and Xu et al. (2023), who concluded that organic inputs can enhance soil enzyme activity and ecological function by increasing organic matter supply and microbial diversity.

### 3.3 Effects of different bacterial fertilizer and green manures on root activity of Korla fragrant pear

This study found that the root activity of Korla fragrant pear was significantly higher in all treatments compared to the control, and the degree of enhancement in-creased with soil depth (0–60 cm). This indicates that the most active root zones were concentrated in deeper soil layers, largely influenced by nutrient availability, moisture, and aeration, which is consistent with the root ecological distribution characteristics proposed by Kang et al. (2022). Among all treatments, JF bacterial fertilizer showed the strongest improvement in root activity at all depths, suggesting that bacterial fertilizer enhances root microecological activity, thereby further promoting root vitality. The effects of green manure treatments followed the order CMX2 > DK1 > CMX1 > DK2, mainly attributed to differences in root exudates, biomass input, and planting density among green manure species, which differentially regulate the rhizosphere environment and soil biological activity. Studies by Nihorimbere et al. (2011) and Yan et al. (2024) have shown that green manure species and planting density synergistically influence the rhizosphere and root metabolic activities, thereby affecting root activity.

In the sweet clover treatment, Morales et al. (2021) reported that the low C/N ratio of sweet clover biomass allows for sustained nitrogen and organic acid release during decomposition, significantly stimulating soil microbial activity and rhizosphere enzyme activities, thereby enhancing root metabolic function. Further, Chelan et al. (2025), Liu et al. (2025), and Kintl et al. (2025) demonstrated that flavonoids, amino acids, and low-molecular-weight organic acids secreted by sweet clover roots not only directly stimulate root activity in Korla fragrant pear, but also significantly increase rhizosphere microbial populations and enzyme activities, accelerating nutrient mineralization and release and forming a positive feedback loop that enhances root activity.

In contrast, Khan et al. (2025) found that moderate biomass input from DK1 helps alleviate rhizosphere oxygen competition and declining aeration while maintaining stable inorganic nitrogen release, thus supporting root activity. However, Araujo et al. (2025) noted that excessive green manure residue input may reduce soil aeration and lead to the accumulation of harmful intermediates, thereby imposing metabolic stress and decreasing root activity.

### 3.4 Effects of different bacterial fertilizer and green manures on root anatomical structure

This study found that both bacterial fertilizer and varying densities of green manure treatments significantly regulated the anatomical structure of Korla fragrant pear roots, as evidenced by a general reduction in cortex thickness across the 0–60 cm soil profile. Tian et al. (2022) demonstrated that organic fertilizer application can improve soil aggregate structure and enhance root metabolic activity and absorption efficiency, which aligns closely with the observed reduction in cortex thickness and enhanced water and nutrient uptake capacity under bacterial fertilizer treatment in this study. Furthermore, Martins et al. (2013) revealed that plants regulate root structure through calcium signaling and green manure–crop rotation systems; the present finding that high-density sweet clover intensified resource competition and triggered adaptive structural adjustments in roots is consistent with this mechanism. Yu et al. (2025) and Zhenggui et al. (2024) also confirmed that different densities and types of green manure have distinct regulatory effects on soil physicochemical properties and crop root structure.

Liu et al. (2010) showed that long-term application of organic and inorganic fertilizers promotes lignin accumulation in roots, thereby increasing mechanical strength. The findings of Ralph et al. (2001) and Chang et al. (2025) indicate that moderate biomass input and balanced carbon–nitrogen dynamics are beneficial for rhizosphere microbial activity and sustained expression of lignin biosynthesis genes. In this study, low-density sweet clover resulted in a greater increase in the proportion of lignified cells in the xylem compared to high-density treatments, which aligns with the aforementioned mechanisms.

Li et al. (2020) and Delphine et al. (2023) reported that soil organic inputs and carbon signaling can induce cell wall thickening and upregulate sugar transporter proteins, while Volker et al. (2009) found that microbial signaling molecules can modulate cell wall microfibril formation and lignin polymerization, thereby enhancing cell mechanical strength and metabolic adaptability. These findings support the present observation that both bacterial fertilizer and green manure treatments promoted phloem structural differentiation in deeper root layers.

Regarding vessel area and section area, Luo et al. (2025) and Luo et al. (2018) confirmed that application of bacterial fertilizer and organic fertilizer enhances root vessel formation and expansion by improving rhizosphere microbial function and hormone synthesis. Consistent with these reports, this study found that both bacterial fertilizer and high-density sweet clover significantly increased vessel area and section area. Additionally, Zhou et al.’s (2023) “channel effect” theory explains the positive influence of oil sun-flower green manure on deep root extension in pear, a result also observed in this study with significant promotion of root depth distribution in the 20–60 cm soil layer under oil sunflower treatment.

### 3.5 Effects of different bacterial fertilizer and green manures on the yield of Korla fragrant pear

The results of this study demonstrate that different types of bacterial fertilizer and green manures significantly increased the yield of Korla fragrant pear. Compared with the control (CK), JF, CMX1, CMX2, and DK2 treatments all effectively improved yield. Previous research by Rad et al. (2022) and Kang et al. (2021) indicated that bacterial fertilizer can promote fruit set and development in fruit trees by improving the root microenvironment, enhancing root activity, and increasing nutrient uptake capacity, which is consistent with the findings of the present study. Among the green manure treatments, sweet clover green manure was particularly effective in enhancing yield. Ma et al. (2021) confirmed that as a leguminous green manure, sweet clover not only possesses nitrogen fixation ability but also markedly improves soil structure, increases soil organic matter and nutrient supply, thereby promoting the growth and yield of fruit trees.

### 3.6 Mantel test and PLS-SEM model

By integrating the Mantel test with Pearson correlation analysis, this study provided an in-depth understanding of the mechanisms by which soil enzyme activities and physicochemical properties influence pear yield and root activity. The results revealed that soil enzyme activities—particularly urease (URE), catalase (CAT), protease (PRO), and nitrate reductase (NR)—were highly and significantly positively correlated with both crop yield and root activity, highlighting the central role of soil enzymes in driving plant root metabolism and yield formation. Enzyme activity directly reflects soil biological function; increased enzyme activity generally indicates heightened microbial activity and accelerated nutrient cycling, thereby supplying more available resources to plants (Wallenstein and Weintraub, 2008). Among these, urease was strongly linked to nitrogen transformation, with its high correlation with yield (*r* = 0.89, *p <* 0.001) suggesting that nitrogen supply efficiency may be a key limiting factor for high yield in pears in the study area (Melara et al., 2025). Similarly, CAT and PRO play important roles in regulating redox balance and alleviating root stress, thereby enhancing root activity and nutrient uptake efficiency (Gianfreda and Rao, 2008). In contrast, correlations between soil physicochemical factors such as pH, EC, and available potassium (AK) and yield or root activity were comparatively weaker, indicating these factors primarily serve as a foundational environment for roots, exerting more indirect effects—mainly by influencing the composition and function of the soil microbial community, which in turn regulates enzyme activity and ultimately impacts plant physiology (Wang et al., 2022). pH was significantly negatively correlated with several enzyme activities, likely because the overall soil was slightly alkaline, and further increases in pH may exceed optimal ranges, suppressing microbial metabolism and plant growth (Estoppey et al., 2025). In addition, root activity showed a significant positive correlation with yield, further emphasizing the key role of root function in supporting high crop productivity. A healthy and active root system not only enhances water and nutrient uptake efficiency but may also improve plant resilience to stress (Philippot et al., 2013). Thus, root activity is not only a prerequisite for yield improvement but also a sensitive indicator of soil management effectiveness (Zhu et al., 2025).

Further structural equation modeling (PLS-SEM) showed that fertilization treatments positively regulated root section structure and root activity—and thus yield—by optimizing soil pH, EC, nutrient levels, and enzyme activities. This is consistent with previous studies showing that fertilization can regulate the root microecology, improving plant nutrient acquisition and root metabolic activity (Nannipieri et al., 2012). Soil nutrient status was not only significantly and positively influenced by soil enzyme activity (*R*^2^ = 0.657), but also exerted the strongest direct effect on root section indices (path coefficient = 0.913), further confirming the pivotal role of soil biological function in crop productivity (van der Heijden et al., 2008). The model also revealed potential nonlinear ecological feedbacks induced by fertilization, such as the negative relationship between soil pH/EC and nutrient supply (path coefficient = −0.527), suggesting that moderate reductions in pH may enhance nutrient availability in the orchard (Zhang et al., 2025). The model exhibited high explanatory power for soil nutrients (*R*^2^ = 0.877), root physiological indices (*R*^2^ = 0.834), and root activity (*R*^2^ = 0.822), with clear structure, robust path fitting, and strong biological significance (Wang et al., 2025). Overall, fertilization mainly drives root and yield responses by regulating soil physicochemical and biological properties, unveiling a multilayered cascade among “fertilization–soil–root–yield” and providing theoretical support for precise fertilization and efficient management (Lai et al., 2025). The structural equation model exhibited clear path relationships and satisfactory explanatory power, supporting both the biological plausibility and statistical reliability of the findings.

## 4 Materials and methods

### 4.1 Overview of the experimental site

This experiment was conducted in Heshilik Township, Korla City, Xinjiang (41°72′78″N, 85°95′46″E, elevation 855.3 m), located in central Xinjiang, on the southern flank of the Tianshan Mountains and the northeastern edge of the Tarim Basin. The region borders the Tianshan foothills to the north and the Taklamakan Desert, the world’s second largest desert, to the south. The area experiences a warm temperate continental climate characterized by large diurnal temperature variation and abundant sunlight, with an annual sunshine duration of 2,990 h. The average annual temperature is 14–15 °C, annual precipitation ranges from 50 to 58 mm, and the maximum annual evaporation is 2,788.2 mm. The region has an effective accumulated temperature of 4,100–4,400 °C, a frost-free period of 210–239 days, and prevailing northeasterly winds.

The soil in the pear orchard is sandy in texture. Basic soil nutrient contents are as follows: organic matter, 11.22 g·kg^−1^; alkaline hydrolysis nitrogen, 16.63 mg·kg^−1^; available phosphorus, 12.06 mg·kg^−1^; available potassium, 167.17 mg·kg^−1^; and pH value, 7.80.

### 4.2 Experimental design

From 2022 to 2023, field experiments were conducted using 7–8-year-old Korla fragrant pear trees (*Pyrus sinkiangensis*), grafted onto *Pyrus betulifolia* rootstock. The experimental orchard was selected based on its long-term uniform management history, ensuring relatively homogeneous soil fertility and tree growth conditions across plots. The planting pattern was 3 m × 5 m, with a planting density of 675 trees per hectare. Six fertilizer treatments were established in the orchard: chemical fertilizer alone, chemical fertilizer plus bacterial fertilizer, and chemical fertilizer plus green manure (various species and sowing methods). Details of fertilizer application rates, green manure species, and sowing techniques are provided in Table 1.

**Table 1:** Experimental processing design scheme.

Experimental processing	Fertilizer type	Sowing amount kg/hm^2^	Sowing depth cm	Row spacing cm	Bacterial fertilizer kg/hm^2^	Nutrient usage kg/hm^2^		
						*N*	P_2_O_5_	K_2_O
CK	Chemical fertilizer	0	0	0	0	300	300	150
JF	Bacterial fertilizer	0	0	0	1,200	300	300	150
DK1	Oil sunflower	27	2–3	25	0	300	300	150
DK2	Oil sunflower	33	2–3	20	0	300	300	150
CMX1	Sweet clover	21	1	25	0	300	300	150
CMX2	Sweet clover	27	1	20	0	300	300	150

Each experimental plot covered an area of 666.67 m^2^ (equivalent to 1 mu), with three replicates per treatment, resulting in a total experimental area of 12,000 m^2^ (18 mu). In autumn, a basal application of 15,000 kg/hm^2^ sheep manure was incorporated once. Phosphorus and potassium fertilizers were applied before spring bud break as single applications: superphosphate (containing 46% P₂O₅) and potassium sulfate (containing 51% K₂O), respectively. Urea (46% N) was used as the nitrogen fertilizer, and the bacterial fertilizer “Shipulang” (produced by Sumitomo Fertilizer, Qingdao), containing *Bacillus subtilis* and *Bacillus licheniformis* with ≥5.0 × 10^8^ CFU/g viable bacteria, was used as the bacterial fertilizer. For both nitrogen and bacterial fertilizer, 60% of the total amount was applied before bud break, and the remaining 40% was top-dressed before fruit enlargement. Green manure was incorporated into the soil at the flowering stage by rotary tillage, following the local orchard practice. After incorporation, irrigation was conducted at intervals of approximately 15–20 days, ensuring favorable conditions for decomposition.

Fertilizer was applied by digging a circular trench 30 cm wide and 30 cm deep at a distance of 50–80 cm from the trunk and uniformly distributing fertilizer within the trench. After fertilization and green manure sowing, conventional orchard management practices were followed, and all treatments were maintained under the same growth and cultivation conditions. Green manure was sown in early April and mowed and incorporated into the soil in late July. The green manure species were sweet clover and oil sunflower, with seeds sourced from Inner Mongolia and purchased from Gansu Lanbin Ecological Technology Co., Ltd. The sweet clover seeds had a purity of 95% and a germination rate of 85%; oil sunflower seeds had a purity of 90% and a germination rate of 80%. The spacing between pear tree rows and green manure strips was maintained at 70–80 cm.

### 4.3 Sampling and Measurement

#### 4.3.1 Sample collection

Soil samples were collected during the fruit enlargement stage of Korla fragrant pear on August 3. For each treatment, soil was sampled from five trees. On both sides of the fertilization trench (5–10 cm from the trench), after removing surface litter, samples were taken from three soil layers (0–20, 20–40, and 40–60 cm). Soil from the same depth on both sides of the trench was combined to form a composite sample per layer. Samples were then preliminarily crushed, homogenized, placed in self-sealing bags, and transported in a dry ice-cooled box to the laboratory. Upon arrival, samples were cleared of roots and large stones, sieved through a 2-mm mesh, and homogenized. Each sample was divided into two portions: one portion, kept on dry ice, was sent for the determination of urease, protease, catalase, and nitrate reductase activities; the other portion was air-dried in the laboratory, sieved through 1-mm and 0.25-mm meshes, and used for soil physicochemical analyses.

Root samples were also collected on August 3 during the fruit enlargement stage, using a root auger (diameter *φ* = 7 cm). For each treatment, roots from five trees were sampled. Along the inner side of the fertilization trench and at four compass points (east, south, west, north) within the tree canopy, after removing surface litter, root samples were taken from three soil depths (0–20, 20–40, 40–60 cm). Roots from the same layer and tree were washed with deionized water and pooled. The cleaned roots were divided into two equal portions: one portion was placed in cryotubes containing FAA (formalin–acetic acid–alcohol) fixative and transported in a dry ice-cooled container to Chengdu Baihui Biotechnology Co., Ltd. for root sectioning; the other portion was brought back to the laboratory for root activity determination. During sampling, pear roots were distinguished from green manure roots by their morphology: pear roots are thicker, yellowish-white to brown, with a developed periderm and distinct branching patterns, whereas green manure roots are much finer and lighter in color. Only pear roots were retained for analysis.

#### 4.3.2 Measurements and calculations

##### 4.3.2.1 Determination of soil physicochemical properties

Soil organic matter content was measured using the external heating method with potassium dichromate and concentrated sulfuric acid. Total nitrogen was determined by the Kjeldahl method after sulfuric acid digestion. Alkaline hydrolysis nitrogen was measured using the alkaline hydrolysis diffusion method. Available phosphorus was extracted with 0.5 mol·L^−1^ NaHCO₃ and determined by the molybdenum antimony colorimetric method. Available potassium was extracted with 1 mol·L^−1^ ammonium acetate and measured by flame photometry. Soil pH was determined with an FE28 pH meter (water:soil ratio 5:1), and electrical conductivity (EC) was measured using a DDS11A conductivity meter.

##### 4.3.2.2 Determination of soil enzyme activities

Urease activity was determined by the sodium phenolate colorimetric method, and its activity was expressed as the milligrams of NH₄^+^–N produced per gram of soil over 24 h. Soil protease activity was measured by the casein colorimetric method and expressed as the milligrams of amino nitrogen produced per gram of soil at 30 °C within 24 h. Catalase activity was determined by the potassium permanganate titration method, and expressed as the volume (mL) of 0.1 mol·L^−1^ KMnO₄ consumed per gram of soil. Nitrate reductase activity was measured by the phenol disulfonic acid colorimetric method, and its activity was calculated as the change in milligrams of nitrate nitrogen per gram of soil before and after reaction.

##### 4.3.2.3 Determination of root activity

Root activity was measured using the 2,3,5-triphenyltetrazolium chloride (TTC) reduction method. First, a standard curve was prepared. Fresh root samples (0.50 g) were weighed and placed into a 25 mL beaker (for the blank, sulfuric acid was added before the root sample; all other procedures were identical). A mixture of 0.4% TTC solution and phosphate buffer (10 mL, equal volumes) was added, ensuring that the roots were fully immersed. The samples were incubated in the dark at 37 °C for 2 h. Then, 2 mL of 1 mol·L^−1^ sulfuric acid was added (except for the blank) to stop the reaction. Roots were removed, blotted dry, and transferred to the original beaker, then extracted with 6 mL of 95% ethanol for 15–20 min (or left overnight until the red pigment was fully extracted). The extract was transferred to a 10 mL centrifuge tube, and the roots were washed 2–3 times with ethanol, with all washings combined in the tube. The final volume was adjusted to 10 mL with 95% ethanol. Samples were centrifuged at 4,000 rpm for 10 min, cooled, and absorbance was measured at 485 nm with a spectrophotometer, using the blank as a reference. The amount of TTC reduction was determined based on the standard curve.

##### 4.3.2.4 Root paraffin sectioning and structural analysis

Root sections were stained using the safranin-fast green method to visualize cell structure and cell wall composition. After fixation in FAA (formalin–acetic acid–alcohol), the root tissues underwent graded alcohol dehydration, clarification with acetic acid, and paraffin embedding. Sections were cut at a thickness of 8 μm, then dewaxed and rehydrated. The staining procedure was as follows: sections were stained in safranin solution for 10 min, rinsed with distilled water, then stained in fast green for 2 min, followed by rapid differentiation in 1% hydrochloric ethanol solution. Finally, sections were dehydrated through graded alcohols, cleared with xylene, and mounted with neutral gum. Under optical microscopy, nuclei and lignified cell walls appeared red, while cytoplasm and celluloserich cell walls were green, clearly displaying the distribution of cell structures and cell wall components in root tissues.

### 4.4 Data processing

All soil physicochemical and enzyme activity measurements were preliminarily processed in Excel 2019. Pearson’s correlation and Waller-Duncan multiple range tests (*p <* 0.05) were performed with IBM SPSS 27.0. Data are expressed as mean ± standard error (x ± se), and figures were drawn in Origin 2022.

Spearman correlation analysis and Mantel tests between enzyme activity, soil properties, and root activity were conducted and visualized using R. PLS-SEM was performed with SMART-PLS. The model was evaluated using standard reliability and validity checks to ensure robustness.

## 5 Conclusion

To improve soil quality and yield while balancing cost reduction, efficiency, and environmentally friendly production, CMX1 is recommended as the optimal management strategy for pear orchards. The findings of this study provide a scientific and technical foundation for the sustainable management of pear orchards in arid regions and broaden the theoretical perspective of sustainable production management. It should be noted that the present study only covers two consecutive years, and therefore reflects short-term field responses. Longer-term trials are still needed to confirm the persistence and stability of the observed effects.”

## Data Availability

The raw data supporting the conclusions of this article will be made available by the authors, without undue reservation.

## References

[ref1] Al-Makhlof H. Mohammed H. Ahmed G. Emhammed M. (2022). Integrated effect of fertilizers on soil pH, EC and organic matter content. J. Pure Appl. Sci. 21, 323–328. doi: 10.51984/jopas.v21i4.2428

[ref2] Amadou A. Song A. Tang Z. X. Li Y. Wang E. Z. Lu Y. Q.. (2020). The effects of organic and mineral fertilization on soil enzyme activities and bacterial community in the below- and above-ground parts of wheat. Agronomy 10:1452. doi: 10.3390/agronomy10101452

[ref3] Araujo A. S. F. Pereira A. P. A. de Medeiros E. V. Mendes L. W. (2025). Root architecture and the rhizosphere microbiome: shaping sustainable agriculture. Plant Sci. 359:112599. doi: 10.1016/j.plantsci.2025.112599, PMID: 40482721

[ref4] Cao W. D. Zhou G. P. Gao S. J. (2024). The role and mechanism of endogenous green manure in driving soil health. J. Plant Nutr. Fertil. 30, 1274–1283. doi: 10.11674/zwyf.2024269

[ref5] Carlson J. Saxena J. Basta N. Hundal L. Busalacchi D. Dick R. P. (2015). Application of organic amendments to restore degraded soil: effects on soil microbial properties. Environ. Monit. Assess. 187:109. doi: 10.1007/s10661-015-4293-0, PMID: 25673270

[ref6] Chang X. Pei Z. Wang X. Wang H. Mu J. Ma Y.. (2025). Divergent responses of plant lignin and microbial necromass to the contribution of soil organic carbon under organic and chemical fertilization. Front. Microbiol. 16:1586791. doi: 10.3389/fmicb.2025.1586791, PMID: 40454367 PMC12124130

[ref7] Chelan Z. A. Amini R. Nasab A. D. M. (2025). Optimizing copper phytoremediation and mung bean yield through *Sinorhizobium meliloti* and *Piriformospora indica* inoculation. Sci. Rep. 15:18759. doi: 10.1038/s41598-025-01681-0 40436908 PMC12120030

[ref8] Dakora F. D. Phillips D. A. (2002). Root exudates as mediators of mineral acquisition in low-nutrient environments. Plant Soil 245, 35–47. doi: 10.1023/A:1020809400075

[ref9] Delphine P. Thomas R. Staffan P. (2023). Cell wall regulation by carbon allocation and sugar signaling. Cell Surface 9:100096. doi: 10.1016/j.tcsw.2023.100096 37396713 PMC10311191

[ref10] Ding B. X. Liu X. Y. He X. F. Chen B. L. Chai Z. P. (2019). Fertilization recommendations for ‘Korla fragrant pear’ orchards based on soil testing. J. Pomol. 36, 1020–1028. doi: 10.13925/j.cnki.gsxb.20180458

[ref11] Drinkwater L. E. Wagoner P. Sarrantonio M. (1998). Legume-based cropping systems have reduced carbon and nitrogen losses. Nature 396, 262–265. doi: 10.1038/24376

[ref12] Ebhin Masto R. Chhonkar P. K. Singh D. Patra A. K. (2006). Changes in soil biological and biochemical characteristics in a long-term field trial on a sub-tropical inceptisol. Soil Biol. Biochem. 38, 1577–1582. doi: 10.1016/j.soilbio.2005.11.012

[ref13] Estoppey A. Michel A. V. Chain P. S. Bindschedler S. Junier P. (2025). Impact of oxalic acid consumption and pH on the *in vitro* biological control of oxalogenic phytopathogen *sclerotinia sclerotiorum*. J. Fungi 11:191. doi: 10.3390/jof11030191 PMC11942934 40137229

[ref14] Gianfreda L. Rao M. A. (2008). Interactions between xenobiotics and microbial and enzymatic soil activity. Crit. Rev. Environ. Sci. Technol. 38, 269–310. doi: 10.1080/10643380701413526

[ref15] He H. Peng M. Hou Z. Li J. (2024). Organic substitution contrasting direct fertilizer reduction increases wheat productivity, soil quality, microbial diversity and network complexity. Environ. Technol. Innov. 36:103784. doi: 10.1016/j.eti.2024.103784

[ref16] Huang J. Hong J. Arango J. Huang D. Huan H. (2025). The response of soil organic nitrogen to the application of green manure mixed with phosphate fertilizer at manure microsite on acidic soil. Agronomy 15:813. doi: 10.3390/agronomy15040813

[ref17] Kang Y. An X. Ma Y. Zeng S. Jiang S. Wu W.. (2021). Organic amendments alleviate early defoliation and increase fruit yield by altering assembly patterns and enzymatic activities in sandy pear. AMB Express 11:164. doi: 10.1186/s13568-021-01322-5 34878599 PMC8655061

[ref18] Kang Y. Ma Y. An X. Kan L. Xie C. Mei X.. (2022). Effects on the root morphology and microstructure of young pear tree by split-root supply of bioorganic and chemical fertilizer. Rhizosphere 22:100504. doi: 10.1016/j.rhisph.2022.100504

[ref19] Kashyap S. Velusamy A. Padhi D. Kumar S. Nayak M. (2025). Exploring the effect of glycerol and low-cost agricultural fertilizer for enhanced microalgae biomass production and wastewater treatment. Algal Res. 90:104117. doi: 10.1016/j.algal.2025.104117

[ref20] Khan A. A. Azeem I. Hui J. Chen Y. Yuan Y. Shah T.. (2025). Non-leguminous green manures improve labile phosphorus availability and crop yield in agroecosystems: a global meta-analysis. Soil Tillage Res. 248:106430. doi: 10.1016/j.still.2024.106430

[ref21] Kintl A. Huňady I. Třináctý J. Richter M. Sobotková J. Hammerschmiedt T.. (2025). Fermentation parameters and nutritional value of silages from fodder mallow, white sweet clover, and their mixtures. Open Agricult. 10:20250435. doi: 10.1515/opag-2025-0435

[ref22] Krishnan G. Holshouser D. Nissen S. (1998). Weed control in soybean with green manure crops. Weed Technol. 12, 97–102.

[ref23] Lai Y. Wang S. Z. Wang Z. Y. (2025). Effects of precision fertilization model on soil nutrients and crop yield of tomato. Agricult. Dev. Equip. 1, 60–62. doi: 10.3969/j.issn.1673-9205.2025.06.020

[ref24] Lan Y. Huang G. Q. Yang B. J. Chen H. J. Wang S. B. (2014). Green manure rotation in rice fields increases soil nutrients and organic carbon pools. Trans. Chin. Soc. Agric. Eng. 30, 146–152. doi: 10.3969/j.issn.1002-6819.2014.13.018

[ref25] Li J. Zhang X. Luo J. Lindsey S. Zhou F. Xie H.. (2020). Differential accumulation of microbial necromass and plant lignin in synthetic versus organic fertilizer-amended soil. Soil Biol. Biochem. 149:107932. doi: 10.1016/j.soilbio.2020.107967

[ref26] Liu N. He H. Xie H. Bai Z. Zhang X. Peng C.. (2010). Impacts of long-term inorganic and organic fertilization on lignin in a Mollisol. J. Soils Sediments 10, 1466–1474. doi: 10.1007/s11368-010-0298-z

[ref27] Liu R. Li C. Zhang Y. Liu C. Xue J. Zheng Y. (2025). Enhanced biological nitrogen fixation and nodulation in alfalfa through the synergistic interactions between Sinorhizobium meliloti and Priestia aryabhattai. World J. Microbiol. Biotechnol. 41:180. doi: 10.1007/s11274-025-04394-8, PMID: 40415123

[ref28] Liu S. Wang Z. Niu J. Dang K. Zhang S. Wang S.. (2021). Changes in physicochemical properties, enzymatic activities, and the microbial community of soil significantly influence the continuous cropping of *Panax quinquefolius* L. Plant Soil 463, 1–20. doi: 10.1007/s11104-021-04911-2

[ref29] Luo B. Hu H. Zheng H. An N. Guo J. Nie Z.. (2025). Fertilization regulates maize nutrient use efficiency through soil rhizosphere biological network and root transcriptome. Appl. Soil Ecol. 207:105912. doi: 10.1016/j.apsoil.2025.105912

[ref30] Luo G. Li L. Friman V. P. Guo J. Guo S. Shen Q.. (2018). Organic amendments increase crop yields by improving microbe-mediated soil functioning of agroecosystems: a meta-analysis. Soil Biol. Biochem. 124, 105–115. doi: 10.1016/j.soilbio.2018.06.002

[ref31] Lyu H. Li Y. Wang Y. Wang P. Shang Y. Yang X.. (2024). Drive soil nitrogen transformation and improve crop nitrogen absorption and utilization – a review of green manure applications. Front. Plant Sci. 14:1305600. doi: 10.3389/fpls.2023.1305600, PMID: 38239220 PMC10794358

[ref32] Ma D. Yin L. Ju W. Li X. Liu X. Deng X.. (2021). Meta-analysis of green manure effects on soil properties and crop yield in northern China. Field Crop Res. 266:108146. doi: 10.1016/j.fcr.2021.108146

[ref33] Martins T. V. Evans M. J. Woolfenden H. C. Morris R. J. (2013). Towards the physics of calcium signalling in plants. Plants 2, 541–588. doi: 10.3390/plants2040541, PMID: 27137393 PMC4844391

[ref34] Melara F. Silva L. K. Mandelli N. A. Krein D. D. C. Chiomento J. L. T. Detmer A.. (2025). Effect on N release by urea coating with chitosan, starch and urease inhibitor. Int. J. Biol. Macromol. 303:140603. doi: 10.1016/j.ijbiomac.2025.140603 39900148

[ref35] Min R. Changjian L. Xiaodong G. Huhu N. Yaohui C. Huixian W.. (2023). High nutrients surplus led to deep soil nitrate accumulation and acidification after cropland conversion to apple orchards on the loess plateau, China. Agric. Ecosyst. Environ. 351:108482. doi: 10.1016/j.agee.2023.108482

[ref36] Morales G. G. Pérez O. J. Manuel J. Yañes S. Vázquez P. A. Castillo F. C. (2021). The sweet clover-*Sinorhizobium meliloti* system as a useful interaction for nitrogen fixation and as a soil improver. Review. Plant Soil 459, 233–246. doi: 10.22319/rmcp.v15i1.6523

[ref37] Mustafa G. Hayat N. Alotaibi B. A. (2023). “How and why to prevent over fertilization to get sustainable crop production” in Sustainable Plant Nutrition, 339–354.

[ref38] Nannipieri P. Giagnoni L. Renella G. Puglisi E. Ceccanti B. Masciandaro G.. (2012). Soil enzymology: classical and molecular approaches. Biol. Fertil. Soils 48, 743–762. doi: 10.1007/s00374-012-0723-0

[ref39] Nihorimbere V. Ongena M. Smargiassi M. Thonart P. (2011). Beneficial effect of the rhizosphere microbial community for plant growth and health. Biotechnol. Agron. Soc. Environ. 15, 327–337. doi: 10.1071/EN10115

[ref40] Ouyang Y. Evans S. E. Friesen M. L. Tiemann L. K. (2018). Effect of nitrogen fertilization on the abundance of nitrogen cycling genes in agricultural soils: a meta-analysis of field studies. Soil Biol. Biochem. 127, 71–78. doi: 10.1016/j.soilbio.2018.08.024

[ref41] Philippot L. Raaijmakers J. M. Lemanceau P. van der Putten W. H. (2013). Going back to the roots: the microbial ecology of the rhizosphere. Nat. Rev. Microbiol. 11, 789–799. doi: 10.1038/nrmicro3109, PMID: 24056930

[ref42] Rad A. K. Zarei M. Astaikina A. Streletskii R. Etesami H. (2022). “Effects of microbial inoculants on growth, yield, and fruit quality under stress conditions” in Sustainable Horticulture, 1–38.

[ref43] Ralph J. Lapierre C. Marita J. M. Kim H. Lu F. Hatfield R. D.. (2001). Elucidation of new structures in lignins of CAD- and COMT-deficient plants by NMR. Phytochemistry 57, 993–1003. doi: 10.1016/S0031-9422(01)00109-1, PMID: 11423146

[ref44] Rao D. Meng F. Yan X. Zhang M. Yao X. Kim K. S.. (2021). Changes in soil microbial activity, bacterial community composition and function in a long-term continuous soybean cropping system after corn insertion and fertilization. Front. Microbiol. 12:638326. doi: 10.3389/fmicb.2021.638326, PMID: 33897643 PMC8059791

[ref45] Shen M. Li J. Dong Y. Zhang Z. Zhao Y. Li Q.. (2021). The effects of microbial inoculants on bacterial communities of the rhizosphere soil of maize. Agriculture 11:50389. doi: 10.3390/agriculture11050389, PMID: 40881048

[ref46] Sorecha E. M. Ruan R. Yuan Y. Wang Y. (2025). Partial substitution of biogas slurry for chemical fertilizer increased wheat grain yield while alleviating N₂O emissions by improving soil quality and regulating N cycling genes. Environ. Technol. Innov. 39:104286. doi: 10.1016/j.eti.2025.104286

[ref47] Stumpf K. Simon C. Miltner A. Maskow T. Lechtenfeld O. J. (2025). Deciphering the energy use channels in soil organic matter: impacts of long-term manure addition and necromass revealed by LC-FT-ICR-MS. Soil Biol. Biochem. 208:109857. doi: 10.1016/j.soilbio.2025.109857

[ref48] Tian S. Zhu B. Yin R. Wang M. Jiang Y. Zhang C.. (2022). Organic fertilization promotes crop productivity through changes in soil aggregation. Soil Biol. Biochem. 165:108533. doi: 10.1016/j.soilbio.2021.108533

[ref49] van der Heijden M. G. Bardgett R. D. van Straalen N. M. (2008). The unseen majority: soil microbes as drivers of plant diversity and productivity in terrestrial ecosystems. Ecol. Lett. 11, 296–310. doi: 10.1111/j.1461-0248.2007.01139.x, PMID: 18047587

[ref50] Volker B. Jane C. S. Shuang W. Wolf-Rüdiger S. (2009). Thaxtomin a affects CESA-complex density, expression of cell wall genes, cell wall composition, and causes ectopic lignification in *Arabidopsis thaliana* seedlings. J. Exp. Bot. 60, 955–965. doi: 10.1093/jxb/ern344 19269997 PMC2652064

[ref51] Wallenstein M. D. Weintraub M. N. (2008). Emerging tools for measuring and modeling the in situ activity of soil extracellular enzymes. Soil Biol. Biochem. 40, 2098–2106. doi: 10.1016/j.soilbio.2008.01.024

[ref52] Wang M. Li Y. Wang Z. Li H. Zuo C. Zhao J.. (2025). Exploring the optical response of water status and light propagation in bruised 'Korla' fragrant pear tissues based on low-field nuclear magnetic resonance coupled with Monte Carlo simulation. Food Chem. 477:143504. doi: 10.1016/j.foodchem.2025.143504, PMID: 39999556

[ref53] Wang C. Ning P. Li J. Wei X. Ge T. Cui Y.. (2022). Responses of soil microbial community composition and enzyme activities to long-term organic amendments in a continuous tobacco cropping system. Appl. Soil Ecol. 169:104210. doi: 10.1016/j.apsoil.2021.104210

[ref54] Wang X. Sun M. Tian L. Yang M. Gao Q. Wang L.. (2025). Microbial fertilizers modulate tobacco growth and development through reshaping soil microbiome and metabolome. Microbiol. Spectr.:e0260524. doi: 10.1128/spectrum.02605-24 40401958 PMC12211045

[ref55] Wang S. J. Tang J. S. Wang J. Yuan K. H. (2025). Estimation and testing of linear regression models from the perspective of SEM. Stat. Decis. 41, 54–58. doi: 10.13546/j.cnki.tjyjc.2025.10.009

[ref56] Xiaomin Y. Karsten S. Yajuan L. Ji M. Reiner D. (2023). Farm size, farmers’ perceptions and chemical fertilizer overuse in grain production: evidence from maize farmers in northern China. J. Environ. Manag. 325:116347. doi: 10.1016/j.jenvman.2022.116347 36244281

[ref57] Xu Y. Ding H. Zhang G. Li Z. Guo Q. Feng H.. (2023). Green manure increases peanut production by shaping the rhizosphere bacterial community and regulating soil metabolites under continuous peanut production systems. BMC Plant Biol. 23:69. doi: 10.1186/s12870-023-04079-0, PMID: 36726076 PMC9890850

[ref58] Xu J. Si L. Zhang X. Cao K. Wang J. (2023). Various green manure-fertilizer combinations affect the soil microbial community and function in immature red soil. Front. Microbiol. 14:1255056. doi: 10.3389/fmicb.2023.1255056, PMID: 38163071 PMC10757628

[ref59] Yan M. Wu M. Liu M. Li G. Liu K. Qiu C.. (2024). Comparative analysis on root exudate and rhizosphere soil bacterial assembly between tomatoes and peppers infected by *Ralstonia*. Chem. Biol. Technol. Agric. 11:36. doi: 10.1186/s40538-024-00561-5

[ref60] Yodphet B. Riddech N. Kaewpradit W. Roytrakul S. Boonlue S. Jangpromma N. (2025). Effect of microbial biofertilizer on proteomic profiling, antioxidant enzyme and andrographolide content in *Andrographis paniculata* under drought stress. Plant Stress 16:100817. doi: 10.1016/j.stress.2025.100817

[ref61] Yu G. Han Y. Liu P. Hao H. Li M. (2025). Response of foxtail millet yield, soil chemical property and bacterial community to different green manure-foxtail millet rotation models in North China. Front. Microbiol. 16:1558354. doi: 10.3389/fmicb.2025.1558354, PMID: 40190739 PMC11968697

[ref62] Zhang Z. He H. Yu M. Xu J. Xu J. Gu J.. (2025). Effects of reduced fertilizer application and soil conditioner on soil acidity, soil nutrients and rice yield. Zhejiang J. Agricult. Sci. 1, 1–9. doi: 10.3969/j.issn.1004-1524.20241073

[ref63] Zhao Y. Wang P. Li J. Chen Y. Ying X. Liu S. (2009). The effects of two organic manures on soil properties and crop yields on a temperate calcareous soil under a wheat–maize cropping system. Eur. J. Agron. 31, 36–42. doi: 10.1016/j.eja.2009.03.001

[ref64] Zhenggui Z. Jian W. Weibin H. Yingchun H. Guoping W. Lu F.. (2024). Respective advantages of growing different green manure with nitrogen fertilization in cotton-based cropping systems: insights from a three-year field study. Food Energy Secur. 13:e70015. doi: 10.1002/fes3.70015

[ref65] Zhou T. Zhang H. Liu Q. Wei L. Wang X. (2023). Effects of fertilizer application patterns on foxtail millet root morphological construction and yield formation during the reproductive stage in the loess plateau of China. Agronomy 13:12847. doi: 10.3390/agronomy13112847, PMID: 40881048

[ref66] Zhu K. Xu Y. Sun Z. Zhang Y. Zhang W. Xu Y.. (2025). Post-anthesis dry matter production and leaf nitrogen distribution are associated with root-derived cytokinins gradient in rice. J. Integr. Agric. 24, 2106–2122. doi: 10.1016/j.jia.2024.02.010

